# A time for sex: circadian regulation of mammalian sexual and reproductive function

**DOI:** 10.3389/fnins.2024.1516767

**Published:** 2025-01-06

**Authors:** Sydney Aten, Oscar Ramirez-Plascencia, Chiara Blake, Gabriel Holder, Emma Fishbein, Adam Vieth, Arman Zarghani-Shiraz, Evan Keister, Shivani Howe, Ashley Appo, Beatrice Palmer, Carrie E. Mahoney

**Affiliations:** ^1^Department of Neurology, Beth Israel Deaconess Medical Center, Harvard Medical School, Boston, MA, United States; ^2^Behavioral Neuroscience, College of Science, Northeastern University, Boston, MA, United States; ^3^Department of Biology, School of Arts and Sciences, Tufts University, Medford, MA, United States; ^4^Department of Psychological and Brain Sciences, College of Arts and Sciences, Boston University, Boston, MA, United States

**Keywords:** circadian, sex, reproduction, mammalian, clock timing, fertility, hypothalamus, hormones

## Abstract

The circadian clock regulates physiological and biochemical processes in nearly every species. Sexual and reproductive behaviors are two processes controlled by the circadian timing system. Evidence supporting the importance of proper clock function on fertility comes from several lines of work demonstrating that misalignment of biological rhythms or disrupted function of the body’s master clock, such as occurs from repeated shift work or chronic jet lag, negatively impacts reproduction by interfering with both male and female fertility. Along these lines, dysregulation of clock genes leads to impairments in fertility within mammals, and disruption of circadian clock timing negatively impacts sex hormone levels and semen quality in males, and it leads to ovulatory deficiencies in females. Here, we review the current understanding of the circadian modulation of both male and female reproductive hormones—from animal models to humans. Further, we discuss neural circuits within the hypothalamus that may regulate circadian changes in mammalian sexual behavior and reproduction, and we explore how knowledge of such circuits in animal models may help to improve human sexual function, fertility, and reproduction.

## 1 Introduction

According to the World Health Organization (WHO), infertility is considered a global public health problem. Indeed, both infertility and subfertility affect a significant proportion of the population: approximately 17.5% of reproductive-aged adults, or ~ 1 in 6 individuals, experience infertility (WHO, 2022). Infertility and subfertility can be divided into several categories with both known and unknown (i.e., idiopathic) etiologies. Many known factors include age at time of conception, ovulatory dysfunction, tubal disease, low sperm count, endocrine, reproductive and/or genetic disorders (Carson and Kallen, 2021; Vander Borght and Wyns, 2018). Notably, 30% of infertile couples, worldwide, are diagnosed with idiopathic infertility, defined as the inability of an otherwise healthy couple (under 35 years of age) to achieve pregnancy after 12 monthly cycles of unprotected intercourse (Sadeghi, 2015).Thus, while the exact causes that drive infertility may depend on several factors, circadian dysfunction likely plays a role in the inability for some of these couples to conceive.

Nearly all organisms rely on the ability to synchronize their physiological and behavioral processes to external time cues. This includes the circadian regulation of sexual behavior and reproduction which is entrained daily to improve the odds of survival for the individual and the species. In mammals, the ‘master clock’ that sustains such intrinsic rhythms is located in the anterior portion of the hypothalamus in a region known as the suprachiasmatic nucleus (SCN). The SCN resides at the top of a hierarchical system consisting of multiple ancillary oscillators found throughout the body and other brain regions. The SCN serves to orchestrate the phase alignment between environmental signals, such as sunrise, with endogenous events, such as peak hormonal release, thus allowing for proper functioning of the hypothalamic–pituitary gonadal (HPG) axis—one of the central regulators of sexual behaviors.

**Figure 1:**
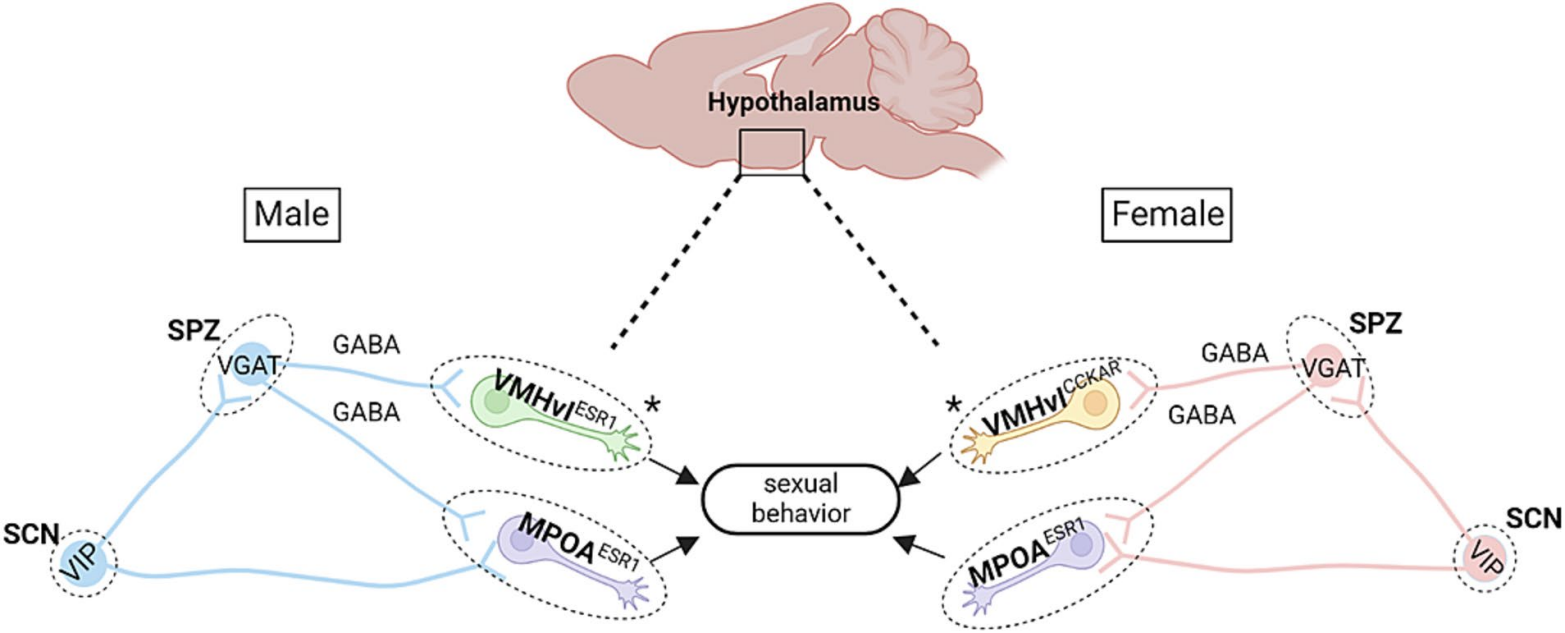
Proposed hypothalamic circuitry modulating time-of-day dependent sexual behaviors in mice. There are several key circuits in the hypothalamus that may be responsible for sexual behavior patterns observed across the day in mice. In both males and females, the subparaventricular zone (SPZ), which contains mainly GABAergic neurons, receives efferent fibers from the SCN. In males, this circuit is further defined by the VMHvl*^Esr1^* cells, wherein low-level stimulation of these cells leads to mounting behaviors (though without USVs; Lee et al., 2014). The SPZ also projects to the mPOA, which mediates sexual motivation (Jennings and de Lecea, 2020). Notably, activation of mPOA*^Esr1^* cells in males leads to USV-positive mounting behaviors and sexual arousal (Karigo et al., 2021). Within females, stimulation of VMHv*l^Cckar^* cells is known to increase female sexual behaviors (Yin et al., 2022), while silencing of mPOA*^Esr1^* cells leads to deficits in female sexual and maternal behaviors (Fang et al., 2018). Asterisks denote the main difference in cell type between males and females. Schematic diagram was created with BioRender.

We discuss the current literature regarding the role of the circadian timing system in the regulation of sexual behavior and reproduction in mammals. To begin, we provide an overview of circadian clock timing and its regulation of both male and female reproductive hormones. We then summarize the role of the SCN and circadian system on conception and outline the hypothalamic neural circuits that have been shown to underlie sexual behaviors. Further, we review circuits and genes in rodent models that may modulate time-of-day dependent changes in sexual behavior(s) (Figure 1), and we highlight how a better understanding of circadian timing and sex can help advance our knowledge of human reproductive behaviors and fertility.

## 2 Overview of the circadian timing system

Nearly every aspect of mammalian physiology and behavior is shaped by a ~ 24 h cycle, or circadian rhythm, which is coordinated by the SCN. This biological timekeeping process is self-sustaining and functions to adjust the body to geophysical time, even in the absence of a zeitgeber, or external timing cue, and it is maintained by circadian clock genes that function to create a transcription-translation feedback loop. From a basic level, at the center of this feedback loop is a basic helix–loop–helix (bHLH) transcription factor heterodimer formed by two proteins: CLOCK and BMAL1. By binding to an E-box enhancer, this activator complex functions to drive the expression of *Period* (*Per1* and *Per2*) and *Cryptochrome* (*Cry1* and *Cry2*) gene families. PER and CRY proteins serve as a heterodimer repressor complex and translocate to the nucleus to inhibit the CLOCK:BMAL1 dimer, thus negatively regulating their own transcription. Degradation of the PER:CRY complex occurs via a phosphorylation and ubiquitin-dependent pathway, and the dissociation of this complex then relieves the repressive effect of the CLOCK:BMAL1 complex to allow for a subsequent round of *Per* and *Cry* gene expression to occur, which generates a ~ 24 h temporal rhythm. For excellent reviews of the mammalian circadian system, we direct reviewers to the following papers: Takahashi (2017) and Lowrey and Takahashi (2000).

Light is the most potent stimulus for entraining intrinsic rhythms of the SCN to the day-night cycle, and the retinohypothalamic tract (RHT) serves as a photic neural input pathway for the SCN to receive light cues (Miyamoto and Sancar, 1998). To this end, the SCN receives light information via intrinsically photosensitive retinal ganglion cells (ipRGCs), which express the photopigment melanopsin, in a monosynaptic connection via the RHT (Hattar et al., 2002; Gooley et al., 2001; Berson et al., 2002; Hattar et al., 2006). These cells release glutamate and pituitary adenylate cyclase-activating polypeptide (PACAP; Hannibal et al., 2000; for an excellent review, please see Morin and Allen, 2006), thus allowing the SCN to transmit this light information to coordinate the phase of circadian oscillators throughout the brain and body (Welsh et al., 2010).

As the master pacemaker of the body, the SCN orchestrates the synchronization of many other oscillator systems. The primary mechanism for such synchronization is thought to be via autonomic neural efferent connections, though non-neural pathways (such as hormones) may also facilitate circadian synchronization (Balsalobre et al., 2000; Vujovic et al., 2008); for details, please see Welsh et al. (2010). Hence, the SCN sends efferent projections to several regions of the brain, including neighboring regions of the hypothalamus (Abrahamson and Moore, 2001; Berk and Finkelstein, 1981; Deurveilher et al., 2002; Deurveilher and Semba, 2005; Kalsbeek et al., 1993; Schwartz et al., 2011), which likely mediate circadian rhythms in sexual and reproductive physiology and behaviors. These brain circuits will be discussed in later sections.

Importantly, it should be noted that the SCN also works as a ‘seasonal clock’, adjusting the general physiology and behavior of the organism to changes in the length of the daytime, including sexual behavior and reproduction. Indeed, key to the evolution of photoperiodism in mammals is the ability to optimize reproductive fitness while also balancing the energy demands needed for sex and survival, which is why photoperiod is important in determining seasonality in many species (Everett and Sawyer, 1950). In organisms that reproduce seasonally, the photoperiod is essential to regulating reproductive capacity, which allows for offspring to be birthed during a time (i.e., season) most suitable for their survival (for review, see Mahoney et al., 2004). This topic of seasonality is beyond the scope of our review; however, we would like to direct readers to several excellent reviews that describe how the SCN regulates photoperiodic information which can subsequently impart control over the neuroendocrine axis, HPG cycle, gonad size, hormone release, and many other downstream processes that modulate seasonal breeding: Brown-Grant and Raisman (1977); de la Iglesia et al. (2003); Mahoney et al. (2004); and Van der Beek et al. (1997).

## 3 Regulation of male reproductive hormones by the circadian system

The mammalian SCN controls daily fluctuations in reproductive hormones. Expression of receptors for numerous reproductive hormones (estrogens, progesterone, and androgens) allows for modulation of the SCN by these hormones (for an excellent review of the neuroendocrinology of the SCN, please see Karatsoreos and Silver, 2007). Of note, while males do possess small amounts of estrogen and progesterone, the focus of this section will be on androgens, as they are sex steroid hormones that regulate the development and maintenance of masculine characteristics via their binding to androgen receptors.

### 3.1 Androgen receptors in the SCN

**Figure 2:**
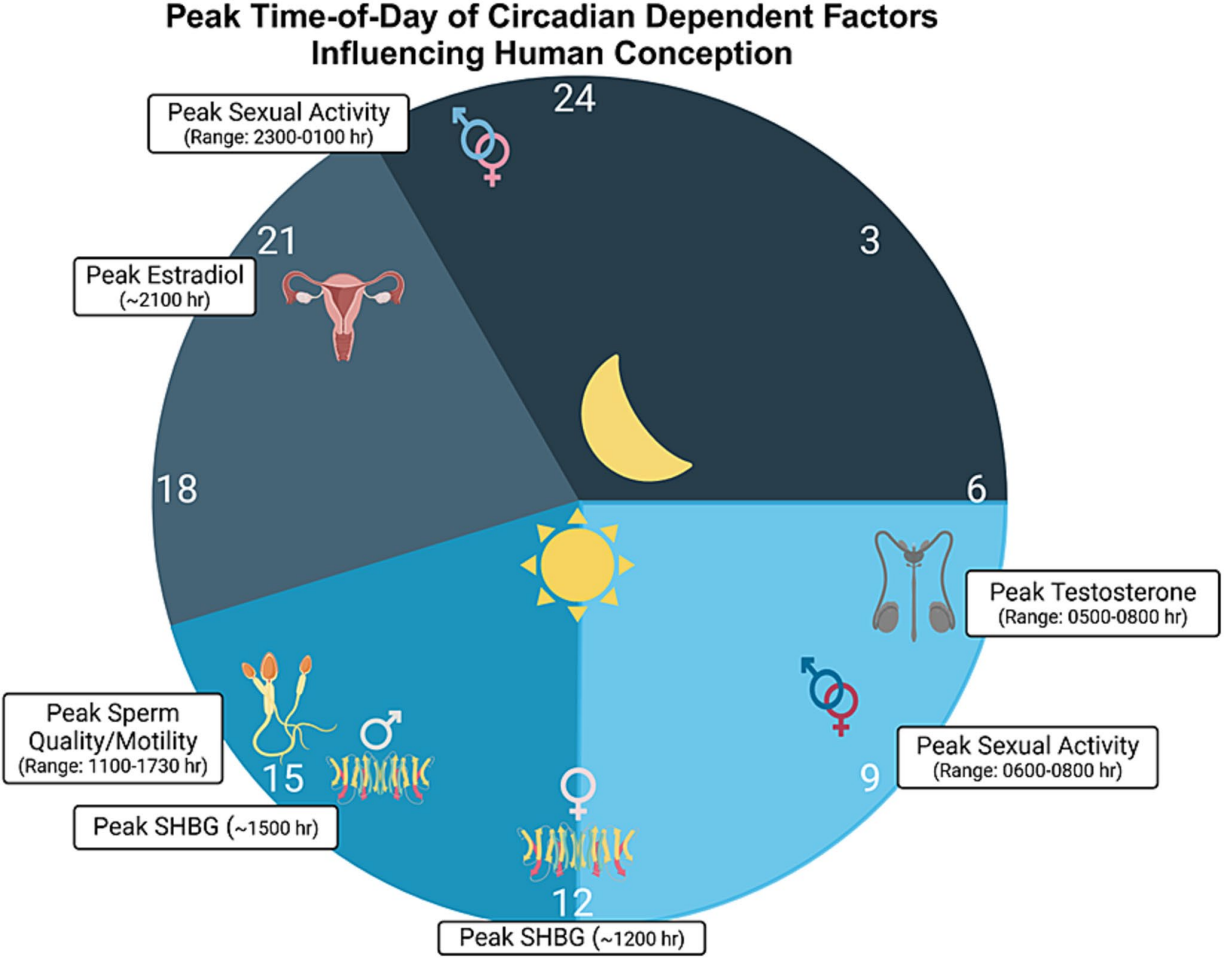
Time-of-day modulated factors influencing conception. Various diurnally-regulated factors likely influence the chances of conception in humans. Such factors include testosterone, with acrophase near 0600 h in males (range: 0500 to 0800 h; de la Torre et al., 1981; Winters, 1991; Diver et al., 2003; Brambilla et al., 2009) and estradiol, with acrophase around 2100 h in women, during the follicular phase (Rahman et al., 2019). Sex hormone binding globulin (SHBG), also known as sex steroid-binding globulin (SSBG), a protein that binds to and helps modulate the availability of sex hormones, has been shown to peak around 1500 in males and 1200 h during the follicular phase in females (Rahman et al., 2019). Additionally, sperm quality and motility are thought to peak between 1100 and 1730 h in males (Cagnacci et al., 1999; Liu K. et al., 2022), with the average peak being around 1430 h. Here, we should note that there are several different reports in the literature regarding the peak time for sperm quality, and the range indicated above is an average, derived from findings in various studies. Finally, many studies have reported peak times for sexual activity in both men and women, with sexual encounters peaking in both the morning and evening (Refinetti, 2005; Palmer et al., 1982b; Fortenberry et al., 2006), often times between 2300 h and 0100 h and with another peak between 0600 h and 0800 h. Sexual activity is denoted by the intertwined male and female sex symbols. Schematic drawing was created with BioRender.

Within the master clock of mammals, androgen receptor (AR) immunoreactive cells have been shown to localize to the SCN core—the main retinorecipient area (Aronin et al., 1990; Rea, 1989). Importantly, in addition to rodents, androgen receptors are found in the SCN of many mammalian species including ferrets, baboons, rhesus macaques, and humans (Fernández-Guasti et al., 2000; Karatsoreos et al., 2007; Kashon et al., 1996; Rees and Michael, 1982; Wu et al., 1995), as described in the following sections. Given that the SCN core is light responsive, and that cells in the SCN core-region contain these ARs, androgen signaling is able to modulate SCN photic responsivity. For instance, Karatsoreos et al. (2007) found that murine SCN cFos expression after a light pulse (both phase-delaying and advancing) is blunted in castrated animals, and replacement with dihydrotestosterone (DHT), a precursor to testosterone, normalized this effect. While reciprocal regulation of androgens and circadian function likely exists (i.e., SCN modulation of androgens and androgen regulation of SCN functionality), in the remainder of this section (and in Figure 2), we will focus on how the circadian system modulates rhythmic changes in testosterone.

### 3.2 Basic research

Testosterone is necessary for sex drive and many other important reproductive functions, such as sperm production (Nassar and Leslie, 2024). Beginning with rodent models, Auer et al. (2020) examined fecal testosterone metabolites excreted from male mice and found that these metabolites showed diurnal fluctuations. Similarly, in young male rats, Esquifino et al. (2004) found a daily rhythm in plasma testosterone, with a peak occurring at approximately zeitgeber time (ZT) 9. Interestingly, the amplitude of this rhythm was blunted in socially isolated rats. Here, it should be mentioned that in many mammals, testosterone is released reflexively during reproduction (Nyby, 2008). Such reflexive pulses occur in two reproductive situations: (1) in response to a sexually arousing stimulus such as a novel female (‘anticipatory’ reflexive pulse) and (2) following ejaculation (‘ejaculatory’ reflexive pulse). In addition to this pulsatile testosterone release, several studies in hedgehogs and rats have also demonstrated an infradian (i.e., longer than 24 h) rhythm in testosterone levels (Diatroptov, 2011; Rutovskaya et al., 2020). Notably, Diatroptov (2011) reported changes in testosterone levels equal to 4 days in male rats, which is the approximate length of the female rodent estrous cycle. Given this, future studies in other species should be conducted to test whether such infradian rhythms in testosterone levels correspond to the mammalian ~4–5 day estrous or ~ 28-day menstrual cycles in females, and if so, whether such an infradian rhythm may contribute to fertility success.

Circadian clock genes play an important role in the circadian regulation of testosterone. For instance, male *Bmal1* knockout mice are infertile and exhibit low testosterone and high LH serum concentrations, suggestive of a defect in testicular Leydig cells (the primary source of testosterone or androgens in males). Importantly, Leydig cells express *Bmal1* in a rhythmic fashion, which suggests that there is peripheral circadian control of testosterone (Alvarez et al., 2008). It is worthwhile to mention, however, that in these studies using *Bmal1* null animals, loss of circadian synchrony within the periphery may be impacting testosterone levels on multiple levels, by reducing production, desynchronizing individual cells, or by blunting rhythms.

### 3.3 Non-human primate research

One of the first published reports of a diurnal rhythm in testosterone came from non-human primate work conducted by Dubey et al. (1983) and Plant (1981). In this study, using rhesus monkeys (a *diurnal* mammal), a continuous light environment was reported to have no effect on the daily rhythm in serum testosterone (Dubey et al., 1983). Additionally, Kholkute et al. (1987) found that a continuous light environment had no effect on the circadian testosterone rhythm of the marmoset—which are also a diurnal species. These results, showing that the rhythms persisted under constant lighting conditions, suggest that testosterone fluctuation is endogenous. However, the role of the rest-activity cycle and photoperiodism versus an endogenous circadian rhythm in diurnal non-human primates is somewhat controversial, given that the nighttime rise in testosterone was abolished when (diurnal) male bonnet monkeys were exposed to constant light conditions (Mukku et al., 1976). In both the Dubey et al. (1983) study and the Mukku et al. (1976) study, animals were exposed to continuous light for up to 15 days. The reason behind these species differences has not yet been elucidated, but the results suggest that testosterone is under the endogenous control of the clock, at least in rhesus monkeys and marmosets. In nocturnal non-human primates such as the owl monkeys and mouse lemurs, testosterone levels were found to be higher during the day (during the rest period) compared to the active (nighttime) period (Dixson and Gardner, 1981; Perret, 1985). These results imply that in some non-human primate species, circadian differences in testosterone levels may correspond with the sleep–wake cycle. Here, it should also be noted that age likely plays a role in the amplitude of the testosterone rhythm given that significant diurnal patterns of testosterone were found in both younger and older rhesus monkeys, with the circadian rhythm being more pronounced in older monkeys (Schlatt et al., 2008).

### 3.4 Human research

Similar to many animal species, in human males, the testes synthesize testosterone—the androgen that is necessary for testicular development and sperm production. Circulating levels of testosterone are modulated by the circadian clock, light exposure, and sleep duration. For example, in healthy young men, serum testosterone concentrations rise with sleep onset, reach the peak during the first REM episode, remain stable until awakening, and then rapidly decline (Luboshitzky et al., 1999). In a similar study, plasma was measured at the following times in 10 men using radioimmunoassay (RIA): 07.00 h, 08.00 h, 09.00 h, 10.00 h, 13.00 h, 16.00 h, 19.00 h, 23.00 h and 03.00 h, and a circadian rhythm was noted, with peak levels occurring between 07.00 h and 10.00 h (Gall et al., 1979). Similarly, a circadian rhythm of testosterone and DHT was detected in three healthy men with a peak at 16.37 (Guignard et al., 1980). However, it is important to distinguish true circadian rhythmicity from sleep–wake effects of variations in testosterone levels. Along these lines, Miyatake et al. (1980) obtained frequent blood samples to measure serum levels of testosterone in healthy men while awake and during sleep, in addition to using electroencephalogram (EEG), electrooculogram (EOG), and electromyogram (EMG) recordings. Results from this study revealed an increase in testosterone during the early morning, and similar results were found using a constant-routine-like paradigm performed on two subjects. This data suggests that such a rise was not directly related to sleep or any specific sleep stages (or from changes in other hormones), but that it is more likely the effect of an endogenous rhythm similar to the rhythm in cortisol (Miyatake et al., 1980). Here, we also direct readers to Figure 2, for a description of other studies that examined circadian rhythms in testosterone levels in men.

The circadian rhythm of other hormones has been reported (for reviews, see Turek et al., 1984 and Kriegsfeld et al., 2006). Indeed, studies have shown that in men, the diurnal pattern of LH and follicle-stimulating hormone (FSH) gonadotropin secretion increases at night and is most pronounced leading up to puberty (for review, see Karatsoreos and Silver, 2007). Furthermore, a study by de la Torre et al. (1981) showed that 17-OH-P and other hormones such as androstenedione and OH pregnenolone showed diurnal variation with highest levels occurring during the morning and lower levels occurring at night.

Circadian and sleep disruption are known to negatively impact reproduction in men. Indeed, several papers have revealed that sleep (a circadian-regulated process) disruption negatively affects testosterone levels and sperm count. For example, during the daytime, Leproult and Van Cauter (2011) found that testosterone levels were decreased by 10 to 15% in young, healthy men who underwent 1 week of restricted sleep (sleeping only 5 h/night). By comparison, it should be noted that normal aging is associated with a decrease in testosterone levels by 0.4–2% annually (Harman et al., 2001; Kaufman and Vermeulen, 2005; Leproult and Van Cauter, 2011; Wu et al., 2008). Whether or not these levels could be normalized upon sleep recovery was not tested in this specific study, though a similar study performed by Arnal et al. (2016) did show that testosterone levels returned to baseline after one night of sleep recovery. Regardless, these studies demonstrate the importance of a functional circadian clock on testosterone levels. Future studies should examine how individual differences in chronotype (Zavada et al., 2005), or how behavioral manifestations of circadian-gated processes such as sleep–wake, diet, activity patterns, etc., affect testosterone production and release in men.

### 3.5 Extrahypothalamic brain structures

Here, it should also be noted that other structures outside of the hypothalamus have AR-immunoreactivity and serve as components of the circadian system and help regulate male sexual and reproductive processes. For instance, in a recently published paper, the activity and expression level of AR in the adult mouse brain was monitored using an AR-Luc reporter mouse. While the main areas of AR immunoreactivity were found to include many regions of the hypothalamus, such as the SCN and the medial preoptic area (mPOA), other regions, such as the amygdala and the stria terminalis, also showed high expression (Dart et al., 2024). Interestingly, a recently paper from the lab of Mark Wu showed that an extra-SCN oscillator in the lateral amygdala expresses the clock-output molecule mWAKE/ANKFN1, and these mWAKE neurons within the amygdala help coordinate rhythmic sensory perception (Liu et al., 2024). Given this, and the fact that amygdala is involved in the regulation of ejaculation (Huijgens et al., 2021) and sexual and aggressive behaviors (Yamaguchi et al., 2020) in male rodents, AR cells within the amygdala may influence the execution of male reproductive behaviors as a function of time-of-day. With respect to the high levels of AR immunoreactivity within the stria terminalis of males (Cara et al., 2021; Dart et al., 2024), it is interesting to note that a neural circuit was recently found to connect chemosensory input with the Bed Nucleus of the Stria Terminalis (BNST)*^Tac1^* neurons. These BNST*^Tac1^* neurons innervate POA*^Tacr1^* neurons and terminate in centers regulating reward during male mating (Bayless et al., 2023).The BNST has been show to exhibit a robust circadian rhythm in the expression of circadian proteins such as PER2 (Amir et al., 2004), suggesting that AR expression within the BNST of males may be involved in mating behavior, drive, and reward across the circadian day.

The pineal gland is another extrahypothalamic structure regulated by the circadian timing system, and AR expression and binding sites have been confirmed in the pineal gland and in pinealocytes of males from many species, including bovine (Haldar and Gupta, 1990), rats (Cardinali et al., 1975), and humans (Luboshitzky et al., 1997). While the pineal gland has many functions, one of its main responsibilities is to receive information regarding the environmental light–dark cycle and to secrete the hormone melatonin (for review, see Arendt and Aulinas, 2000; Sapède and Cau, 2013). Furthermore, the pineal gland has been shown to exert a prominent role in the neuroendocrine regulation of reproductive physiology (Aleandri et al., 1996; D’Occhio and Suttie, 1992; Li and Zhou, 2015). For example, pineal hormones like melatonin regulate reproduction in seasonal animals such that adult male hamsters exposed to short photoperiods exhibit gonadal regression (Mason et al., 2010). Additionally, the presence of melatonin receptors in the sperm of several species (Casao et al., 2012), humans included (van Vuuren et al., 1992; Espino et al., 2011), suggests that it may directly modulate the ability of sperm to fertilize an egg to form a zygote. While a detailed discussion of melatonin and male reproduction is outside the scope of this paper, we direct readers to an excellent review on this topic: Li and Zhou (2015).

## 4 Regulation of female reproductive hormones by the circadian system

Estradiol (E2) and progesterone (P) are steroid hormones responsible for the development of female sexual characteristics, the maintenance of the reproductive cycle, and pregnancy in females. Hence, this section will focus on the circadian regulation of E2 and P given that they are important in driving female sex and reproduction by binding to estrogen and progesterone receptors (ERs, such as ERα and ERβ, and PR).

### 4.1 Estrogen and progesterone receptors in the SCN

Several studies have shown the presence of ERs and PR within the SCN. High levels of ERβ and low levels of ERα are expressed in the SCN of neonatal rats. Notably, these results differ from work conducted in adult rats which failed to show ERα expression and showed only low levels of ERβ expression in the SCN. Of note, both receptor subtypes are expressed in neurons as well as in astrocytes, and some cells express both ERα and ERβ (Su et al., 2001). With respect to the topographic distribution of ERα and ERβ in the SCN, the shell region displays high levels of ER-immunoreactivity, while the SCN core shows little expression (Vida et al., 2008). Interestingly, PR expression varies across species, though it is present in the SCN of humans, and ERα and ERβ are maintained in the SCN of humans, though ERα expression is stronger than ERβ (Yaw et al., 2020; Kruijver and Swaab, 2002). Such differential distribution of ERs within the master circadian clock suggests several modes of estrogenic signaling within the SCN (and between the SCN and other extra-SCN brain regions), which may influence the circadian modulation of certain reproductive-related events and pathways. Notably, most studies have examined how estrogens act on non-SCN, ER-rich regions of the brain that receive innervation from the SCN (directly or indirectly), including the mPOA, amygdala, and ventromedial hypothalamus (VMH; Laflamme et al., 1998). Along these lines, many neural efferent fibers from the SCN project to the SPZ (Leak and Moore, 2001; Watts, 1991; Watts and Swanson, 1987), and the SPZ projects to many of these above-listed ER-rich brain regions (Vujovic et al., 2015). Furthermore, some of these ER-rich regions may project to and modulate the SCN, and thus, the estrogenic effects on circadian rhythms may also arise from indirect actions of estrogens on target regions of the SCN. These data suggest that ER signaling may affect clock timing or tau length, but this should be confirmed experimentally. The remainder of this section will focus on daily rhythms in estrogens and circadian control of estrogenic signaling.

### 4.2 Basic research

Daily oscillations in ER expression have been reported and may contribute to the circadian regulation of estrogen-dependent behaviors. For example, a study of mice in constant darkness (DD), revealed a circadian pattern of ER𝛽 expression which remained intact after 3 days in DD (Cai et al., 2008). ER𝛽 mRNA levels were also shown to fluctuate in various peripheral tissues, with a peak occurring at the light–dark transition. Notably, this study also found that this rhythm was abolished in clock-deficient *Bmal1* knockout mice, and when CLOCK and BMAL were introduced *in vitro*, ER𝛽 expression increased. These findings not only indicate that ER𝛽 expression oscillates in a circadian fashion, but also that this oscillation is mediated by endogenous clock genes (Cai et al., 2008). Taken together, these studies show that both ERα and ER𝛽 both exhibit circadian patterns of expression which occur as a result of proper core clock gene expression/function.

The circadian system modulates female reproductive behaviors in various ways, and one example is its regulation of the estrous cycle—the ~4–5-day reproductive cycle in rodents which consists of four distinct phases: proestrus, estrus, metestrus, and diestrus. Ovulation (which typically occurs during the night of estrus) in female rodents is very complex and requires tight temporal control from cells spanning from the SCN, to gonadotropin-releasing hormone (GnRH) neurons within the hypothalamus, in addition to temporal control of many other hormones (for an excellent review, please see Miller and Takahashi, 2014). In brief, elevated levels of E2 are necessary for the GnRH surge to occur, during the late proestrus phase of the cycle. This GnRH signal triggers the surge of LH and promotes FSH release from the pituitary gland. Within the ovaries, LH induces ovulation and FSH initiates the recruitment of new follicles (Miller and Takahashi, 2014). Notably, in addition to the GnRH surge, a time-dependent signal must also occur to induce the preovulatory GnRH surge (Everett and Sawyer, 1950). The SCN was shown to control this neural time cue (i.e., the preovulatory hormone surge) given that SCN dysregulation leads to acyclicity of the estrous cycle in rats (Brown-Grant and Raisman, 1977; Terasawa et al., 1980; Wiegand and Terasawa, 2008).

How the SCN regulates this circadian neural timing signal leading to the preovulatory GnRH surge and subsequent induction of LH has not been completely elucidated. However, two peptides within the SCN–vasopressin (AVP) and vasoactive intestinal polypeptide (VIP)—likely regulate the temporal release of GnRH. Along these lines, GnRH neurons are directly innervated by VIPergic projections from the SCN (Kriegsfeld et al., 2002; van der Beek et al., 1993), and VIP administration has also been shown to stimulate the LH surge (Samson et al., 1981). Additionally, AVP administration modulates the LH surge such that it occurs in the late afternoon in rats (Palm et al., 2001). Furthermore, inhibition of either peptide in rats results in a decrease in the amplitude of the E2-induced LH surge (Funabashi et al., 1999; Harney et al., 1996; van der Beek et al., 1999, and for an excellent review, also see Russo et al., 2015). Studies performed on GT1-7 neuronal cells (a mature mouse hypothalamic GnRH line) revealed that high levels of E2 leads to circadian expression of the kisspeptin peptide receptor GPR54 *in vitro* (Tonsfeldt et al., 2011). Notably, the SCN can also regulate circadian GnRH release indirectly via kisspeptin-expressing neurons in the anteroventral periventricular nucleus (AVPV; Van der Beek et al., 1997). Kisspeptin-expressing neurons in this region have been shown to play a relevant role in the regulation of GnRH secretion in rodents (see Skorupskaite et al., 2014, for review). The SCN targets AVPV kisspeptin neurons via AVP projections (Vida et al., 2010), thus inducing a circadian rhythm in the response of GnRH to kisspeptin (Williams et al., 2011). Hence, it is possible that the SCN regulates the GnRH surge by direct innervation via AVP and/or VIP, and by an indirect pathway through AVPV kisspeptin neurons. For an excellent review of this topic, see Miller and Takahashi (2014). In another line of work, a study by Vieyra et al. (2016) demonstrated that for ovulation to occur in rats, the system modulating GnRH and LH secretion requires a cholinergic signal arriving on either the right or left SCN on the morning of proestrus. This study reported that cholinergic innervation arriving on either side of the SCN may also help modulate progesterone and estradiol secretion according to time-of-day. These experiments may also help to explain why unilateral SCN injury (which would block this cholinergic innervation to the SCN) is sufficient to decrease the number of shed ova in rats (Ramírez et al., 2017). Taken together, these studies suggest that a complex interplay between SCN neuronal output and kisspeptin receptors within the AVPV are necessary for the preovulatory GnRH surge in females.

Evidence of the importance of a functional master circadian clock in the regulation of this LH surge comes from studies showing that *Clock* mutant rats lack a coordinated LH surge on the proestrus day, and they exhibit disrupted estrous cycles. *Clock* mutant rats also show a high rate of full-term pregnancy failure and a decrease in P levels during pregnancy (Miller et al., 2004). Additionally, SCN injury has been shown to affect hormone levels and ovulation, as bilateral SCN injury in rats resulted in anovulation and, as noted above, (unilateral) right SCN injury led to fewer ova being shed (Ramírez et al., 2017). In these studies, however, it is important to consider that many key circadian genes (such as *Clock*) are transcription factors that influence the expression of thousands of downstream genes, and thus, their pleiotropic effects must be considered. In a similar fashion, older techniques, such as SCN lesion and/or injury often destroy many other brain tissues, some of which may be involved in reproduction, which must also be taken into consideration. As such, future studies that employ targeted deletion of clock gene expression specific to certain cell types, will shed light on circadian control of female ovulation. For an excellent review that highlights a role for the clock in steroid hormone synthesis, ovarian follicular growth, and ovulation, we direct readers to Sellix (2015).

### 4.3 Human research

In humans, several components that modulate sexual and reproductive behaviors appear to be rhythmic. This is the case for ERs, given that, in a study of human mammary cell lines, Rossetti et al. (2012) reported that ERα mRNA oscillates in a circadian fashion in ERα-positive breast epithelial cells. Furthermore, Bao et al. (2003) found a daily rhythm in free E2 in cycling women, with a peak occurring in the early morning. More recently, Rahman et al. (2019) also noted a significant circadian rhythm in plasma E2, P, and other hormones under both standard sleep–wake cycle and in constant routine conditions during the follicular phase. Interestingly, only two hormones, FSH and sex hormone binding globulin (SHBG) were rhythmic in the luteal phase, suggesting differential circadian control depending on menstrual cycle phase (i.e., pre- versus post-ovulation). In another study by Fujimoto et al. (1990), 7 of 10 cycling women exhibited a significant circadian variation in P during the luteal phase, though the acrophase of such a rhythm was not consistent among the 7 women. Although it was not accounted for in the study, the individual differences in tau may have contributed to the differences in peak expression. We also direct readers to Figure 2, for a depiction of findings related to circadian rhythms in E2 and P.

Circadian clock disruption in women leads to adverse effects on hormones that regulate reproduction and fertility. Along these lines, several studies have shown that women working night shifts, or those who have irregularly scheduled shifts, display an increase in menstrual pain and changes in menstrual bleeding (Chung F. F. et al., 2005; Labyak et al., 2002). Specifically, Lawson et al. (2011) showed that women with over 20 months of rotating shift work were more likely to experience irregular cycles, and cycles were more likely to be inconsistent in length. Several other studies demonstrated that such menstrual changes also included irregular patterns of ovarian and pituitary hormone secretion (Chung K. et al., 2005; Lohstroh et al., 2003, and for excellent reviews, please see Knutsson, 2003; Mahoney, 2010; Scott, 2000). On a similar note, it has been reported that female university students exhibit higher menstrual symptoms, pain, behavioral changes, and water retention scales when their social jet lag is greater than 1 h (Komada et al., 2019). Furthermore, women who partake in transmeridian travel showed a reduction in sleep, in addition to fatigue and insomnia (for review, please see Sack, 2009)—factors that likely impact hormone secretion. Indeed, it is known that sleep deprivation alters LH amplitude and E2 concentrations (Baumgartner et al., 1993), and thus, circadian disruption stemming from jet lag and/or shift work is likely a key factor in endocrine and reproductive dysfunction observed in women who consistently work irregular hours/night shifts and in those who consistently travel. This topic will be discussed in greater detail in the last section.

### 4.4 Extrahypothalamic brain structures

Similar to the Male section (3) above, other regions of the brain, outside of the hypothalamus, have ER and P-immunoreactivity. Extrahypothalamic expression of ERα are highly expressed in the BNST, the amygdala, and regions of the locus coeruleus and periaqueductal gray (Mitra et al., 2003; Shughrue et al., 1998), while cells with high ER𝛽 expression are found in the lateral septum, BNST, and amygdala (Creutz and Kritzer, 2002; Milner et al., 2010; Mitra et al., 2003; Shughrue et al., 1998). For an excellent review of ERs in the CNS of females, we direct readers to the following article: Almey et al. (2015). High extrahypothalamic expression of PRs within the female brain are observed within regions of the frontal cortex and hippocampus (Guerra-Araiza et al., 2002) and the BNST and amygdala (Auger and De Vries, 2002; Kato et al., 1994; for review, see Brinton et al., 2008). Of these noted brain structures, the BNST and amygdala are also known to express circadian clock genes and modulate female sexual and reproductive behaviors. For example, in the BNST and central nucleus of the amygdala, PER2 protein rhythms peak early in the dark phase (Amir et al., 2004; Lamont et al., 2005). Yamazaki et al. (2000) recorded multiple unit neural activity from both in and outside of the SCN of golden hamsters and found that SCN and BNST rhythms were always in-phase, suggesting a strong coupling between the two regions. Concerning, the regulation of sex and reproductive function, Martinez and Petrulis (2011) found that, in female Syrian hamsters, the BNST is important for the normal expression of sexual solicitation behaviors in response to male odor. Similarly, the medial amygdala is necessary for opposite-sex odor preference and vaginal marking (Petrulis and Johnston, 1999), in addition to two other female reproductive behaviors: ultrasound production and lordosis (Kirn and Floody, 1985; Rajendren and Moss, 1993; for review, see Sakuma, 2015).

As in males, the pineal gland is another extrahypothalamic structure that is involved in both circadian clock timing and in the regulation of female reproductive processes. The female rat pineal gland contains both ERα and ER𝛽 subtypes (Sànchez et al., 2004), and PR expression has been noted in the bovine pineal gland (Vacas et al., 1979). Interest in the role of the pineal gland in the regulation of female reproduction has grown significantly in recent years given its role in producing melatonin. Indeed, Kennaway and Rowe (1997) found that female rats treated with melatonin showed inhibition of ovarian development and delayed puberty onset, suggestive of melatonin’s involvement in modulating ovarian growth and functionality. Melatonin levels in follicular fluid (from women) has also been shown to vary indirectly with day length and with P levels, (Rönnberg et al., 1990; Yie et al., 1995), and such variations indicate that melatonin could alter female reproduction in humans prior to ovulation. There is a significant positive correlation between melatonin in follicular fluid and follicle count in women undergoing *IVF* which suggests that melatonin may also provide a protective role during the ovarian cycle (Zheng et al., 2018). However, high dose melatonin, when combined with P, is able to inhibit ovulation in women (Voordouw et al., 1992). For a detailed description of the role of melatonin in female reproduction, we direct readers to two excellent reviews: Olcese (2020) and Fernando and Rombauts (2014). These studies demonstrate that the pineal gland—via its ability to modulate the circadian clock and secrete melatonin—is a significant player in female reproduction.

## 5 Regulation of conception by the circadian system

### 5.1 Role of the SCN and circadian system in conception—with a focus on males

The role of the circadian clock and its modulation of factors that influence conception in males is less well studied compared to the female estrous cycle/ovulation. Notably, however, the quality of semen has been shown to change diurnally in human males. Indeed, semen samples have been shown to have the highest levels in sperm concentration in the early morning (Xie et al., 2018). However, this is in contrast to a study in which seminal fluid was collected by masturbation (twice by each subject–once in the morning and once in the afternoon). Indeed, in this latter study, Cagnacci et al. (1999) also showed the number of spermatozoa with linear motility was higher in the afternoon than the morning and that although macroscopic parameters were similar, specimens collected in the afternoon showed a higher number and concentration of sperm. It should be highlighted though, that of these 54 enrolled males, 24 were normozoospermic and 30 suffered from oligo-and/or asthenozoospermia. Notably, in their experiments, subjects were randomized to have sperm collected in the morning or afternoon after 3 days of abstinence, such that the time between the previous ejaculation and time of morning or afternoon collection was consistent among each subject (Cagnacci et al., 1999). Here, we should also report that a daily diurnal variance in sperm DNA fragmentation index has been shown in both humans and in mice, with a nadir occurring at 10 AM (Ni et al., 2019).

The importance of a proper timekeeping system in the regulation of male factors that influence conception is reinforced by studies showing the effects that the disruption of clock genes can have on various aspects of male reproduction. Perhaps the most striking evidence comes from the fact that male mice with homozygous mutations in *Clock* and *Bmal1* have reduced fertility (Alvarez et al., 2008; Dolatshad et al., 2006; Liang et al., 2013). In addition to these reductions in fertility, changes in testosterone function have also been reported (see the Male Reproductive section). *Bmal1* null mice also have alterations in testes physiology given their decreased average seminiferous tubule diameter (Alvarez et al., 2008), and they have alterations in the structure of chromatid bodies of their spermatids (Peruquetti et al., 2012). It should also be noted that abnormalities in testicular function have been found in *Cry1* knockout mice, wherein deficiency of this gene increases germ cell apoptosis within the testes of mice and decreases sperm count (Li et al., 2018). Interestingly, Morse et al. (2003), found that constant expression levels of *Per1* and *Bmal1* were observed within the testis of mice but another study found that *Per3* expression did exhibit prominent circadian rhythms within the mouse testes (Zylka et al., 1998), suggesting that circadian clock machinery can drive testicular function (Baburski et al., 2016; Alvarez et al., 2008), particularly within Leydig cells, which produce testosterone. For an excellent overview of the complexity of the rhythmic functions of cells within the testis, we direct readers to Bittman (2016).

These aforementioned studies provide strong support for the necessity of proper circadian functioning in conception. However, as noted previously, in the studies that used mice deficient in circadian clock genes, the pleiotropic effects of such genes must be considered, given that clock genes target a variety of downstream factors that can also influence conception and pregnancy viability. On a separate note, interestingly, while restricted feeding during either the day or nighttime period was found to disrupt rhythms in male mouse mating behaviors (Kukino et al., 2022), no study (to date) has examined whether a rhythm exists in sexual behavior within mice housed in constant conditions (and in mice fed *ad libitum*). Given that mice are used as a model organism for dissection of neural circuits, future studies should test whether a sexual behavior rhythm occurs and if so, whether this rhythm may coincide with a rhythm of conception.

### 5.2 Role of the SCN and circadian system in conception—with a focus on females

In female mice, ovulation occurs during the morning of estrus, after the proestrus LH surge, and if mating (with a male) occurs, copulation allows for circadian-gated prolactin (PRL) surges (Freeman and Neill, 1972; Mai et al., 1994; Poletini et al., 2010; for an excellent review, please see Miller and Takahashi, 2014). To this end, retinorecipient neurons within the SCN release VIP which provides an inhibitory stimulus to the dopaminergic neurons within the hypothalamic periventricular-arcuate nucleus (Pe). PRL release will continue for a period of ~10 days following conception or up to 12 days following a pseudopregnancy (Freeman et al., 1974). Also modulated by the circadian clock is the LH surge that promotes ovulation (as described in the section above; Mosko and Moore, 1979; Williams et al., 2011; Williams and Kriegsfeld, 2012). Such clock-gated events of hormonal release must occur in females to increase the odds of successful conception. Furthermore, circadian genes play an important role in the circadian regulation of conception since female mice lacking functional *Bmal1* or *Per1* or *Per2* all show deficiencies in embryonic implantation and/or maintenance of pregnancy. For an excellent review depicting the role of clock genes in pregnancy maintenance, please see Miller and Takahashi (2014).

Implantation, which, depending on the species, occurs hours to weeks after fertilization, is the process by which the fertilized egg(s) implants in the female uterus to allow the embryo(s) to grow and develop. Interestingly, to date, no studies have been conducted on the timing of embryo transfer (into the uterus) during assisted reproduction (such as *in vitro* fertilization). However, circadian clock gene expression has been observed in female reproductive structures that influence implantation: such as the uterus (Uchikawa et al., 2011; Johnson et al., 2002) oviduct (Johnson et al., 2002; Kennaway et al., 2003), ovarian granulosa cells (Mereness et al., 2016) and embryo (prior to implantation; Johnson et al., 2002). Notably, several studies have found that *BMAL1* null female mice experience implantation failure (and subsequent infertility) due to a reduction in progesterone production (Ratajczak et al., 2009; Liu et al., 2014). Given this, it may be worthwhile to examine whether the time-of-day of embryo transfer impacts implantation success. One could postulate that transfer of embryos into the uterus during a specific time of day may allow for an increased chance of implantation, possibly due to circadian regulation of steroidogenesis and endometrial thickness/receptivity (Ratajczak et al., 2009; Liao et al., 2022). Such data could have a profound impact on assisted reproductive technology advancement.

## 6 Proposed circadian neuronal circuits that control physiology and sexual behavior to optimize conception

In 1970, Dr. Curt P. Richter found that, in rats, successful mating depends on the presence of estrus (in the female rat) in addition to proper functioning of the 24-h circadian clock of both the male and the female. Indeed, Richter performed an experiment that involved blinding rats (to remove all external light timing cues) and found that pregnancy could only be achieved when the active phases of the circadian clocks in both the male and the female rat overlapped, when the female was in estrus. This was the first study to report a circadian rhythm in sexual behavior within mammals (Richter, 1970). Around the time of Dr. Richter’s study, it was becoming increasingly well-known that circadian rhythms in physiology and behavior arise from the SCN (Moore and Eichler, 1972; Ralph et al., 1990; Saper, 2013; Hastings et al., 2018). Studies on mechanisms as to how the SCN maintains the daily synchrony of such processes demonstrated that temporal control highly depends on its efferent targets and the related functions of these target areas. Many of these SCN targets are also seated in the hypothalamus, which houses structures that are known to control sexual behaviors (for reviews, see Calabrò et al., 2019; Iovino et al., 2019). In this section, we will focus on two such brain regions: the VMH and the mPOA. However, we will first discuss how the subparaventricular zone (SPZ) serves as a relay between the SCN and these ‘downstream’ hypothalamic structures.

### 6.1 Subparaventricular zone

The SPZ is dorsally adjacent to the SCN in the anterior portion of the hypothalamus, and it runs in a dorsal-posterior arc just below the paraventricular hypothalamus (thus giving it the name ‘subparaventricular zone’). The SPZ receives the densest efferent fibers from the SCN and is thought to be a key relay center for driving rhythms in physiology and behavior (Leak and Moore, 2001; Watts, 1991; Watts and Swanson, 1987). To this end, lesioning the SPZ or cutting through it with a knife (Inouye and Kawamura, 1979; Lu et al., 2001; Schwartz et al., 2009) attenuates rhythms in locomotion and behavior. Ibotenic acid lesions to the ventral SPZ (vSPZ) in rats impairs the circadian rhythm of sleep and locomotor activity under constant conditions. Lesions to the dorsal SPZ (dSPZ) lead to the immediate loss of circadian rhythmicity of body temperature, even when rats are placed in light/dark conditions (Lu et al., 2001), and more recently, a viral-based technique that renders SPZ cells unable to release GABA, resulted in the loss of aggression rhythms in male mice (Todd et al., 2018). Such results, coupled with the fact that the SPZ shows a consistent phase relationship to the SCN (Inouye and Kawamura, 1979; Kubota et al., 1981; Nakamura et al., 2008; Sato and Kawamura, 1984), support the idea that the SPZ serves as a major conduit for output signals emanating from the SCN. Importantly, the SPZ consists of mainly GABAergic neurons (Lein et al., 2007) which are found within its regionalized subdivisions: namely, dorsal-medial, dorsal-lateral, ventral-medial, and ventral-lateral (Costa et al., 1999; Gooley et al., 2001; Johnson et al., 1988; Leak et al., 1999; Leak and Moore, 2001; Levine et al., 1991; Lu et al., 2001; Moore et al., 2002; for review, see Aton and Herzog, 2005; Canteras et al., 2011). In a study published by Vujovic et al. (2015), the differences in projections from these various SPZ sub-compartments were examined in mice using both anterograde and retrograde tracing techniques. They found that the SPZ projects to the basal forebrain, pons and brainstem, thalamus, habenula, cortex, and hypothalamus, including the mPOA. The dorsolateral SPZ also shows extremely dense projects to the dorsomedial and central VMH. Given that the SPZ serves as a key output of the SCN (as noted above), and that efferent fibers from the SPZ project to both the mPOA and VMH, these results raise the prospect that a circuit comprising the SCN→SPZ→VMH or SCN→SPZ→mPOA may regulate time-of-day dependent sexual behaviors, two topics discussed below.

### 6.2 Ventromedial hypothalamus

For the past several decades, the VMH has been known to be an integral player for many neuroendocrine functions within mammals, including sexual behavior. First, in Mathews and Edwards (1977) lesioned discrete areas of the hypothalamus and determined the VMH was involved in the regulation of sexual behavior within female rats given that these lesions eliminated sexual receptivity. Later in the same decade, researchers determined the VMH was critical in regulating female lordotic behavior (Goy and Phoenix, 1963; Pfaff and Sakuma, 1979b, Pfaff and Sakuma, 1979a), and in males, it was shown to modulate scent marking and partner preference, though limited effects on mounting behaviors were noted (Harding and McGinnis, 2003, 2005).

With respect to subtypes of neurons within the VMH, it is important to consider the fact that many VMH neurons are sexually dimorphic (Yang et al., 2013). Though a detailed depiction of sexual dimorphism is outside the scope of this review, we direct readers to several excellent, recently published articles that describe sexual dimorphic subpopulations within the VMH: Cortes et al. (2023) and Khodai and Luckman (2021). In brief, PR number, distribution, and projection(s) vary by sex within the mouse brain, and in female mice, ablation of PR-expressing neurons in the ventrolateral division of the ventromedial hypothalamus (VMHvl) diminished sexual receptivity, while in male mice, it led to deficits in mating behaviors (Yang et al., 2017). In addition to PR, a recent study also showed that cholecystokinin A receptor (*Cckar*)-expressing cells within the VMHvl are key regulators of female sexual behaviors. Indeed, inactivation of these cells in female mice decreases their interest in males and decreases female sexual receptivity, and activating these cells increases their sexual behavior (Yin et al., 2022). These results, coupled with the fact that female mice lacking *Cckar* show deficits in female-specific sexual behaviors (Xu X. et al., 2012), raise the prospect that VMHvl*^Cckar^* neurons could potentially control circadian-dependent female sexual behaviors.

Interestingly, VMH circuitry drives both aggression and sexual behaviors. Indeed, optogenetic stimulation of VMHvl neurons positive for ERα—a nuclear receptor and transcription factor derived from the *Esr1* gene, has been shown to increase male mounting behaviors at low-intensity stimulations, while higher intensity stimulation was shown to increase aggressive behaviors (Lee et al., 2014; Lin et al., 2011). However, it should be noted that male mice emit ultrasonic vocalizations (USVs) when courting female mice (for review, see Egnor and Seagraves, 2016), and a recently published paper indicated that male mounting evoked by such weak activation of VMHvl*^Esr1^* neurons actually represents a form of aggression (Karigo et al., 2021). One study conducted in the lab of Clif Saper, demonstrated that there exists time-of-day dependent (i.e., circadian) aggression behavior in male mice (Todd et al., 2018). While the SCN weakly projects to the VMH, Todd et al. (2018) found an SCN relay through the dorsal SPZ to the VMHvl*^Esr1^* neurons, which influences time-of-day dependent aggression behaviors. Hence, it is possible that the same pathway also modulates circadian dependent sexual behaviors in males and females (see Figure 1).

### 6.3 Medial preoptic area

The mPOA is another candidate brain region that may modulate time-of-day dependent sexual behaviors in both males and females (for excellent reviews regarding the mPOA and sexual behavior, see Hull and Dominguez, 2007; Hull and Rodríguez-Manzo, 2009; Rodríguez-Manzo and Canseco-Alba, 2017). The mPOA receives a wide range of afferent input, especially from the olfactory system and genitals (Hull and Dominguez, 2007). Damage to the mPOA impairs sexual behavior in rodent models of both sexes (Christensen et al., 1977; De Jonge et al., 1989; Gray and Brooks, 1984; Hansen, 1982; Klaric and Hendricks, 1986; Larsson and Heimer, 1964; Liu et al., 1997; for review see Paredes and Baum, 1997) and stimulation of the mPOA enhances sexual behaviors (Malsbury, 1971; Rodríguez-Manzo et al., 2000; for review see Paredes, 2003).

Similar to the VMH, the mPOA is also one of the most widely-accepted sexually dimorphic brain regions. Indeed, the mPOA differs between males and females in most species (humans included; Allen et al., 1989; Hofman and Swaab, 1989; Swaab and Fliers, 1985; Xu Z. et al., 2012), and various genes within this brain region may be responsible for circadian-dependent sexual behaviors. Interestingly (as briefly noted above), it has been shown that in male mice, the mPOA*^Esr1^* hypothalamic subpopulation is enriched with neurons that induce a reproductive-like state within males, characterized by USV-positive vocalizations when stimulated, while stimulation of VMHvl*^Esr1^* neuronal subpopulations promote USV-negative mounting and likely represent an aggressive-like state (Karigo et al., 2021). Hence, in male mice (and likely in female mice too), a circuit spanning the SCN–SPZ–mPOA*^Esr1^* may regulate time-of-day dependent sexual behaviors. However, it is important to note that these mPOA*^Esr1^* are characterized by different subpopulations involved in either parenting and/or mating. Along these lines, a transcriptomic study conducted by Moffitt et al. (2018) reported that, while 6 different subpopulations are enriched in males after mating, only 2 are enriched in females. Thus, it is important to keep in mind that this circuitry seems to be quite complex, especially when interpreting which *Esr1* subpopulation is being directly regulated by the circadian system. Consistent with the idea that a circuit from the SCN to mPOA modulates circadian sexual behavior, Schwartz et al. (2011) demonstrated a direct projection exists from the SCN to the mPOA. Their results also show that projections of both the SCN and the vSPZ are conserved in both diurnal and nocturnal rodents. Vujovic et al. (2015) also reported modest projections from the dSPZ to the mPOA within mice (Vujovic et al., 2015). Hence, a direct or indirect pathway from the SCN→mPOA may be regulating circadian sexual behaviors. Identification of the cell population chemotype involved with circadian regulation of sexual behavior will be critical as Ribeiro et al. (2012) showed that viral-vector mediated RNA interference in female mice, used to silence ERα/ESR1 expression specifically in the mPOA, leads to deficits in maternal care and sexual behavior. The fact that mPOA*^Esr1^* cells play crucial roles in maternal behaviors in female mice (Fang et al., 2018), coupled with the fact that the mPOA expresses some of the highest levels of *Esr1* within the brain (Mitra et al., 2003; Shughrue et al., 1997), advances the prospect that an SCN–SPZ–mPOA*^Esr1^* circuit could also modulate circadian dependent sexual behaviors (Figure 1).

## 7 Significance to human sexual behavior and reproduction

### 7.1 Time-of-day differences in human sexual behavior

While there is mixed literature as to what time-of-day men and women typically desire to have sex, a circadian variation in sexual activity has been reported (also see Figure 2). For example, a study of 78 young married couples demonstrated a major peak in sexual activity in the evening and another peak in the morning (Palmer et al., 1982a). A study by Refinetti (2005) drew similar results, showing two large peaks of sexual encounters among a study conducted on 15 university students. One peak occurred between 11:00 PM and 1:00 AM, and the second peak occurred between 6:00 AM and 8:00 AM. In another study, it was found that coital events in adolescents were most likely to occur after 6 PM compared to other times of day (Fortenberry et al., 2006). However, it is likely that the major reason for this timing choice was partner availability. This idea is supported by a study conducted by Jankowski et al. (2014), which showed that the timing of the desire of sexual activity is only modestly positively associated with the actual timing of when the sex takes place. Hence, while rhythms in reproductive hormones and chronotype of both sexual partners presumably plays a role in sexual behavior across the day (Jocz et al., 2018), it is likely that temporal restrictions brought upon by social engagements has a significant effect on what time-of-day humans choose (and most desire) to engage in sexual intercourse. Future studies aimed at teasing apart the precise time-of-day as to when men and women are most sexually aroused (in the absence of social or light timing cues) would be of interest. Such a study would help determine whether sexual behavior is truly modulated in a ‘circadian’ manner within humans. Further, testing whether such a peak in sexual desire may coincide or overlap with a peak time-of-day for conception may be an interesting future study, especially with regards to maximizing fertility, described below. Along these lines, it would be worthwhile to identify whether having sexual intercourse in the morning (when male testosterone levels are highest) leads to better chances for an ovulating female to conceive. Additionally, whether sexual behavior may serve as a zeitgeber for men and women would be an interesting area of inquiry to further understand the interaction of circadian rhythmicity and sexual activity.

### 7.2 Circadian dysfunction function and human fertility and reproduction

Large scale assessment of gene expression with spatial transcriptomics has the potential to illuminate coordinated molecular relationships, not only by spatial, functional or pathological themes, but by temporal dimension (Zhang et al., 2023). Researchers have been able to highlight genetic underpinnings of male infertility (Llavanera et al., 2022; Singh Jamwal, 2023; Liu Q. et al., 2022) and female reproductive function, such as expression of the proliferative marker MKI67 (Rubel et al., 2012; Knoedler et al., 2022). The contribution of circadian control over or across multiple cell types, spatial relationships, functional synchrony and pathological states is critical to dissecting the complexity of successful reproduction. As described by Rubel et al. (2012) with their results of microarray analysis from uterine cells from mice, the role of progesterone in circadian rhythm gene expression in the uterus may have profound indications of multi-levels of circadian influence on peripheral reproductive organs. We anticipate that genes regulating the priming of reproductive organs, such as *Wnt7A* to support the endometrium, and hyaluronan binding protein 4 involved in chromatin remodeling during male germ cell development, to support health of sperm (Sutovsky and Lovercamp, 2010; Sutovsky et al., 2024; Zhang et al., 2023), will be under circadian control, and their respective apex will occur prior to peak sexual behavior. Investigation into proper temporal alignment between central and peripheral reproductive structures will provide great insight for therapy or treatment of infertility and/or reproductive organ dysfunction. Furthermore, central influence of sex hormones may regulate cell type-specific transcriptome to modulate sexual behavior (Knoedler et al., 2022).

Circadian disruption, most often due to rotating and night shift work, leads to irregular menstrual cycles and hormone abnormalities (as noted previously in this review). Such disruption has also been shown to increase the risk of pre-term birth and lead to a decrease in fecundity in women (Lawson et al., 2011; Nurminen, 1998; for excellent reviews, see Baker and Driver, 2007; Reschke et al., 2018). Epidemiological studies have also shown that maternal chrono-disruption leads to other adverse pregnancy outcomes such as smaller fetus size (for the gestational age) and an increased chance for miscarriage and spontaneous abortions (Abeysena et al., 2009; McDonald et al., 1988; Whelan et al., 2007; Zhu et al., 2004; for review, see Knutsson, 2003). Furthermore, genetic polymorphisms in the core circadian clock genes *Npas2* and *Bmal1* are known to contribute to adverse fertility outcomes (Kovanen et al., 2010). Future studies should examine clock gene expression changes in older women (>35 yrs) trying to conceive, and whether chrono-disruption, which negatively impacts a host of biological processes including sleep, temperature, and blood pressure, is more detrimental (in terms of fertility and pregnancy outcomes) in women >35 yrs. trying to become pregnant. Such information could prove beneficial in advising women who wish to conceive.

With regards to dysfunction of the circadian timing system on male reproduction, in addition to alterations in testosterone levels and rhythm following chrono-disruption (described earlier in this review), several studies have teased apart the influence of circadian desynchrony on spermatogenesis. For example, a statistically significant correlation between sleep duration and testis volume has been reported (Zhang et al., 2018). Importantly, sleep is one of many circadian-modulated processes that may confound circadian disruption of reproductive function. Furthermore, in a meta-analysis, Zhong et al. (2022) showed that sleep disorders were associated with reduced sperm count, reduced sperm concentration, and reduced sperm morphology and motility. Interestingly, bioinformatic mining in this same study revealed that circadian clock genes *Per1, Per2, Cry2, Nr1d1,* and *Npas2* were also decreased in males who lacked sperm in ejaculate (azoospermia; Zhong et al., 2022). Similar genetic variations have been reported in men experiencing infertility. For example, a homozygous mutation in *Npas2* has been reported in a Turkish family with nonobstructive azoospermia (Ramasamy et al., 2015a). Additionally, variability within the *Clock* gene is also associated with reduced semen quality and idiopathic infertility in men (Hodžić et al., 2013; Shen et al., 2015; Zhang et al., 2012). Such studies raise the prospect that alterations in clock genes may serve as a molecular marker for male infertility, and they highlight the fact that therapeutic interventions aimed at stabilizing circadian rhythms may be potential treatments for cases of male infertility. These results also raise interesting questions regarding the ability to modulate clock timing to improve fertility in aging men. Along these lines, though females are typically thought to have a ‘ticking biological clock’, studies suggest that increasing male age is also associated with a significant decline in fertility (for reviews, see Pasqualotto et al., 2008; Ramasamy et al., 2015b). Hence, gaining a better understanding of how changes in clock genes might signal the alarm of declining fertility in aging men may prove of value to couples trying to conceive.

### 7.3 Summary

Overall, gaining a better understanding of how circadian rhythms modulate male and female hormones, sexual behavior, and ultimately reproduction will prove fruitful in improving fertility outcomes, particularly in those individuals struggling to conceive. Future studies that aim at investigating which brain circuits control such sexual and reproductive behaviors as a function of time-of-day will help to advance our knowledge of mammalian fertility and could provide new avenues for therapeutic interventions and drug targets for fertility medications.
